# Relationship between healthy habits and sociodemographic variables and risk of diabetes type 2

**DOI:** 10.4314/ahs.v22i4.17

**Published:** 2022-12

**Authors:** Ángel Arturo López González, Pere Riutord Sbert, Bartomeu Riutord Fe, Neus Riutord Fe, Sebastiana Arroyo Bote, José Ignacio Ramírez Manent

**Affiliations:** 1 Department of Odontology, Faculty of Odontology, ADEMA University School. Palma. Spain; 2 Balearic Islands Health Service. Palma, Spain

**Keywords:** Diabetes mellitus, physical activity, Mediterranean diet, social class

## Abstract

**Background:**

Type 2 diabetes is considered a worldwide public health problem due to its high prevalence and the important complications it causes.

**Objectives:**

To assess the influence of healthy habits, especially physical activity and diet, on the risk of type 2 diabetes.

**Material and methods:**

Descriptive and cross-sectional study in 1457 Spanish workers in which the influence of different sociodemographic variables and lifestyle habits such as tobacco consumption, physical activity and adherence to the Mediterranean diet in relation to the risk of presenting type 2 diabetes assessed with the Finrisk and Leicester scales was evaluated.

**Results:**

The prevalence of moderate and high values of the Finrisk and Leicester scales decreased as the level of physical activity assessed with the IPAQ questionnaire increased and as adherence to the Mediterranean diet increased. In the multivariate analysis by binary logistic regression, high age, male sex, less favoured social class, low level of physical activity and low adherence to the Mediterranean diet influence the increase in the prevalence of high diabetes risk values, while tobacco consumption shows a protective effect.

**Conclusion:**

Physical exercise and the Mediterranean diet have a beneficial effect on the risk of presenting type 2 diabetes with Finrisk and Leicester scales.

## Introduction

Type 2 diabetes is one of the diseases with the greatest social and health impact due to its high prevalence, chronic complications and high mortality rate^1^. In the year 2019, approximately 9.3% of the world's adult population suffered from diabetes, and according to estimates, by the year 2045 the prevalence will increase to 10.9%, which constitutes a significant increase in the number of people affected by this disease in the coming years^2^.

Diabetes is a chronic systemic disease with a complex pathogenesis characterized by peripheral insulin resistance, abnormal regulation of hepatic glucose production and decreased beta-cell function, which ultimately leads to beta-cell failure. Among its complications we can highlight retinopathy, nephropathy and neuropathy, the latter being the most frequent complication and affecting 40% of diabetics^3^.

The probability of developing type 2 diabetes depends on a combination of risk factors, including genes and lifestyle. Some of these factors, such as family history, age or race, cannot be modified, but those related to diet, physical activity and weight can be influenced. These lifestyle changes can influence the likelihood of developing type 2^4^ diabetes. The inclusion of regular physical activity is critical for optimal insulin action and glycemic control in individuals with diabetes. Current research suggests that type II diabetes mellitus is preventable and that all types of diabetes can be controlled with physical activity, largely through improvements in muscle sensitivity to insulin^5^. There are different scales to assess the risk of type 2 diabetes, the most widely used being the Finrisk^6^ which has been validated in a large number of countries, and there are also others such as QDSCORE^7^, Leicester^8^ or Ausdrik^9^.

The aim of this study was to assess the influence of the Mediterranean diet, physical exercise and sociodemographic variables such as sex, age and social class on the risk of type 2 diabetes as determined with the Finrisk and Leicester scales.

## Methods

Retrospective and cross-sectional study of 1584 workers in the Balearic Islands and the Valencian Community carried out between January 2017 and December 2017. A total of 127 were excluded (69 did not accept to participate and 58 due to ages not included in the study) leaving 1457 workers who are the ones finally included in the study, of whom 718 were women (mean age 43.30 years) and 739 were men (mean age 46.02 years). The workers were selected from among those who attended periodic occupational medical check-ups.

### Inclusion criteria

- Age between 18 and 67 years.

- To be an active worker.

- Belonging to one of the companies collaborating in the study.

- Accepting to participate in the study.

The anthropometric, clinical and analytical determinations were performed by the health personnel of the different occupational health units participating in the study, after homogenizing the measurement techniques.

For the measurement of weight, which is expressed in kilograms, and height, which is expressed in cm, a scale with a measuring rod is used: SECA model 700. The abdominal waist circumference (in cm) is measured with a measuring tape: SECA model 20 with the person in a standing position, feet together and trunk straight, abdomen relaxed and upper limbs hanging on both sides of the body. The tape measure is placed parallel to the floor at the level of the last floating rib. Hip circumference is measured with the same tape measure and adopting the same position as for the waist circumference and passing the tape horizontally at hip level. The waist/height and waist/hip indices are obtained by dividing the waist circumference by the height and hip circumference respectively. The cut-off point for the former is 0.50 and for the latter 0.85 for women and 0.95 for men^10^.

Blood pressure was obtained in the supine position with a calibrated OMRON M3 automatic sphygmomanometer and after 10 minutes of rest. Three measurements are taken at one-minute intervals and the mean of the three is obtained. Blood tests are obtained by peripheral venipuncture after a 12-hour fast. Samples are sent to reference laboratories and processed within 48–72 hours. Automated enzymatic methods are used for blood glucose, total cholesterol and triglycerides. Values are expressed in mg/dl. HDL is determined by precipitation with dextran sulfate Cl2Mg, and values are expressed in mg/dl. LDL is calculated using the Friedewald formula (provided that triglycerides are less than 400 mg/dl). Values are expressed in mg/dl.

Friedewald formula: LDL= total cholesterol -HDL- triglycerides/5

The glycemia figures were classified according to the recommendations of the American Diabetes Association^11^; patients with a previous diagnosis, those who after obtaining a glycemia figure higher than 125 mg/dL presented a glycosylated hemoglobin≥ 6.5% or if the person was taking hypoglycemic treatment were classified as diabetic. A smoker was considered to be a person who had regularly consumed at least 1 cigarette/day (or the equivalent in other types of consumption) in the last month, or had quit smoking less than a year ago.

For social class, we used the 2011 National Classification of Occupations (CNO-11) and the proposal made by the social determinants group of the Spanish Society of Epidemiology^12^. We chose to classify into 3 categories: Class I. Directors/managers, university professionals, athletes and artists. Class II. Intermediate occupations and self-employed workers without employees. Class III. Unskilled workers.

Diet is assessed by means of the questionnaire on adherence to the Mediterranean diet^13^ which is based on the Predimed test and consists of 14 questions, each scored with 0 or 1 point. Scores below 9 are considered low adherence and above 9 good adherences.

Physical activity is determined by means of the International Physical Activity Questionnaire (IPAQ)^14^. This is a 7-question self-administered questionnaire that assesses the type of physical activity performed in daily life during the last 7 days.

### Test Value

1.Walking: 3′3 METx minutes of walking x days per week.

2.Moderate Physical Activity: 4 MET*x minutes x days per week

3.Vigorous Physical Activity: 8 MET*x minutes x days per week.

The three values obtained are then added together

CLASSIFICATION CRITERIA:

Moderate Physical Activity:

- 3 or more days of moderate physical activity and/or walking at least 30 minutes per day.

- 5 or more days of any combination of walking, moderate or vigorous physical activity achieving at least a total of 600 METs.

Vigorous Physical Activity:

- Vigorous Physical Activity at least 3 days per week achieving a total of at least 1500 METs.

- 7 days of any combination of walking, moderate physical activity and/or vigorous physical activity, achieving a total of at least 3000 METs.

MET is the Unit of Measurement of the test

Findrisk model (Finnish Diabetes Risk Score) estimates the probability of developing type 2 diabetes in the next ten years^15^. The questionnaire contains eight questions and scores range from 0 to 26 points. The variables included in the questionnaire are: body mass index, age, sex, waist circumference, physical activity, diet (daily intake of fruits and vegetables), personal history of elevated blood glucose or antihypertensive treatment and family history of diabetes. From 12 points it is considered moderate and from 15 points high.

The Leicester Diabetes Risk Score determines the probability of developing type 2 diabetes in the next ten years^8^. The questionnaire consists of eight questions and scores range from 0 to 41 points. The variables included in the test are: body mass index, age, sex, race, waist circumference, personal history of elevated blood glucose, personal history of elevated blood pressure or antihypertensive treatment, and family history of diabetes. From 7 and above is considered medium risk and from 16 and above high risk.

### Statistical analysis

A descriptive analysis of the categorical variables was performed, calculating the frequency and distribution of responses for each of them. For quantitative variables, the mean and standard deviation were calculated, and for qualitative variables, the percentage was calculated. The bivariate association analysis was performed using the 2 test (with correction of Fisher's exact statistic when conditions required it) and Student's t test for independent samples. For the multivariate analysis, binary logistic regression was used with the Wald method, with calculation of the Odds ratio and the Hosmer-Lemeshow goodness-of-fit test. Statistical analysis was performed with the SPSS 27.0 program, with an accepted statistical significance level of 0.05.

### Ethical considerations and aspects

The study was approved by the Clinical Research Ethics Committee of the Illes Balears health area no. IB 4383/20. All procedures were performed in accordance with the ethical standards of the institutional research committee and with the 2013 Declaration of Helsinki. All patients signed written informed consent documents before participating in the study.

## Results

The values of the anthropometric, clinical, analytical, sociodemographic and healthy habits variables in our population are more unfavourable, except for total cholesterol, among men. The complete data are presented in Table 1.

**Table 1:** Characteristics of the population

	Women (n=718)	Men (n=739)	Total (n=1457)	

	mean (SD)	mean (SD)	mean (SD)	p-value
**Age (years)**	43.30 (8.44)	46.02 (8.50)	44.68 (8.57)	<0.0001
**Height (kg)**	66.29 (12.29)	82.24 (13.81)	74.38 (15.32)	<0.0001
**weight (m)**	1.62 (0.06)	1.73 (0.07)	1.68 (0.09)	<0.0001
**BMI (kg/m^2^)**	25.36 (4.61)	27.40 (4.13)	26.39 (4.49)	<0.0001
**Waist (cm)**	89.44 (16.36)	97.00 (10.65)	93.27 (14.27)	<0.0001
**Hip (cm)**	105.78 (13.22)	108.77 (10.27)	107.29 (11.91)	<0.0001
**Systolic Blood** **Pressure (mm Hg)**	121.31 (17.05)	133.76 (18.11)	127.62 (18.66)	<0.0001
**Dyastolic Blood** **Pressure (mm Hg)**	75.03 (10.58)	80.63 (11.43)	77.87 (11.36)	<0.0001
**Cholesterol (mg/dl)**	186.02 (31.14)	183.37 (31.72)	184.67 (31.46)	0.108
**HDL (mg/dl)**	60.18 (13.55)	49.83 (12.16)	54.93 (13.86)	<0.0001
**LDL (mg/dl)**	107.88 (28.16)	108.94 (29.15)	108.42 (28.66)	0.483
**Triglycerides (mg/dl)**	86.57 (43.59)	119.55 (87.42)	103.30 (71.28)	<0.0001
**Glycaemia (mg/dl)**	92.16 (16.31)	98.68 (19.54)	95.47 (18.30)	<0.0001

	%	%	%	**p-value**

**<35 years**	16.71	10.42	13.52	<0.0001
**35–49 years**	57.80	51.01	54.36	
**≥ 50 years**	25.49	38.57	32.12	
**Social class I**	18.94	8.80	13.80	<0.0001
**Social class II**	63.65	82.67	73.30	
**Social class III**	17.41	8.53	12.90	
**No tobacco**	71.87	72.94	72.41	<0.0001
**Yes tobacco**	28.13	27.06	27.59	
**MET low**	23.68	19.08	21.35	<0.0001
**MET moderate**	48.05	36.4	42.14	
**MET high**	28.27	44.52	36.51	
**Predimed low**	36.49	48.17	42.42	<0.0001
**Predimed high**	63.51	51.83	57.58	

The prevalence of moderate and high values of the two types 2 diabetes risk scales (Finrisk and Leicester) decreases as the level of physical activity increases, this can be observed in both sexes, see Table 2.

**Table 2:** Prevalence of altered values of the different metabolic and diabetes risk scales according to physical activity by gender

	Women				Men			

	MET low	MET moderate	MET high		MET low	MET moderate	MET high	

	n=170	n=345	n=203		n=141	n=269	n=329	

	%	%	%	p-value	%	%	%	p-value
**Finrisk moderate-high**	17.90	10.29	5.94	<0.0001	34.75	16.33	8.49	<0.0001
**Leicester risk moderate-** **high**	17.90	17.06	9.09	0.001	44.62	38.25	27.04	<0.0001

Something similar occurs with the prevalence of moderate and high values of these scales in persons with high adherence to the Mediterranean diet as shown in Table 3.

**Table 3:** Prevalence of altered values of the different metabolic and diabetes risk scales according to healthy food by gender

	Women			Men		

	Predimed low	Predimed high		Predimed low	Predimed high	

	n=262	n=456		n=356	n=383	
	%	%	p-value	%	%	p-value
**Finrisk moderate-high**	14.79	8.50	<0.0001	21.79	12.09	<0.0001
**Leicester risk moderate-** **high**	18.29	13.42	0.012	35.82	32.97	0.064

Male sex, age over 50 year s, tobacco use, low or moderate physical exercise, low adherence to the Mediterranean diet, and social classes II and III were used as covariates. The variable that most increases the risk of presenting moderate or high values of the two types 2 diabetes risk scales is age after 50 years, followed by social class. Tobacco consumption will increase the risk of presenting type 2 diabetes with the two scales analysed. All the results are presented in Table 4.

**Table 4:** Logistic regression analysis

	Men	≥50 years	Smokers	MET low- moderate	Predimed low	social class II- III
**Finrisk moderate-high**	1.45 (1.02–2.05)	6.34 (4.48–8.97)	1.21 (1.08–1.35)	2.88 (1.91–4.33)	2.19 (1.56–3.08)	3.14 (1.52–6.49)
**Leicester risk moderate-high**	2.88 (2.12–3.91)	9.70 (7.24–12.99)	1.37 (1.15–1.60)	1.80 (1.31–2.49)	1.43 (1.06–1.91)	3.31 (1.85–5.91)

## Discussion

In our study, the prevalence of moderate or high values of the Finrisk and Leicester scales decreased as the level of physical activity and adherence to the Mediterranean diet increased. In the multivariate analysis, all the covariates analysed in the study (male sex, age 50 years and older, more disadvantaged social classes, low level of physical activity, low adherence to the Mediterranean diet, and tobacco use) increased the risk of presenting type 2 diabetes. Practically all the studies consulted agree with the results obtained by us, although they do not always use the same risk scales or assess physical activity or diet as we have done. Many studies have been carried out in adults and some in younger populations than ours.

There is abundant epidemiological and clinical evidence showing that physical activity is associated with a reduction in the prevalence of cardiovascular disease, hypertension, metabolic syndrome and type 216 diabetes. This beneficial effect of exercise on the appearance of diabetes was also found, specifically on the risk of its appearance using one of the questionnaires administered in our study, in a research study carried out in almost a thousand Turkish adults^17^. Crump et al. worked along the same lines, finding an inverse association between objectively measured muscular and cardiorespiratory fitness and the risk of incidence of type 2 diabetes. This work included nearly 1.5 million 18-year-old soldiers who did not have type 2 diabetes at baseline, with long-term follow-up into adulthood. Both cardiorespiratory and muscular fitness were independently associated with the risk of type 2 diabetes incidence, and the combination of low cardiorespiratory fitness and muscular fitness increased the risk of developing type 2 diabetes by 3 times. The major limitation of this work is that it was only performed in men^18^. Other research has focused more on the beneficial effect of exercise on glycemic control^19^.

Other studies have evaluated the joint effect of physical exercise and diet, but not focusing on the Mediterranean diet as we did, and have observed positive results on diabetes, both in terms of risk and clinical control. Regarding the effect on the risk of developing type 2 diabetes some structured lifestyle intervention trials involving moderate intensity physical activity at least 150–175 min/week and dietary energy restriction targeting weight loss of 5%-7% demonstrated 40%-70% reductions in the risk of developing type 2 diabetes in people with impaired glucose tolerance^20^.

A 2015 systematic review evaluating 66 lifestyle intervention programs (physical activity and diet) concluded that they reduced the incidence of type 2 diabetes, body weight and blood glucose, while also improving other cardiometabolic risk factors^21^. Other studies that evaluated less intensive lifestyle interventions have also demonstrated efficacy in reducing diabetes^22^.

Mera-Gallego et al in 2017 found results similar to ours in a population of 628 Spanish adolescents in whom the aim was to identify risk factors for developing type 2 diabetes determined by means of the Findrisk questionnaire modified for adolescents, the results revealed an inverse correlation between the Findrisk score and the values adherence to a healthy diet assessed with the Kidmed index and physical activity with the IPAQ-A questionnaire^23^.

Among the strengths of this study are the large sample size (almost 1500 persons), the application of a scale little used in our setting such as the Leicester scale, and the fact that the assessment of physical activity and adherence to the Mediterranean diet was carried out with validated questionnaires (IPAQ and Predimed).

The main limitation of the study is that it was carried out in a very specific geographic area, which may make it difficult to extrapolate the results to other countries.

## Conclusions

Regular physical activity and adherence to the Mediterranean diet decrease the risk of developing type 2 diabetes. Age over 50 years and belonging to social classes II-III are the risk factors that most increase the probability of developing type 2 diabetes.
